# The Mutational Road not Taken: Using Ancestral Sequence Resurrection to Evaluate the Evolution of Plant Enzyme Substrate Preferences

**DOI:** 10.1093/gbe/evae016

**Published:** 2024-01-30

**Authors:** Emily M Catania, Nicole M Dubs, Shejal Soumen, Todd J Barkman

**Affiliations:** Department of Biological Sciences, Western Michigan University, Kalamazoo, MI 49008, USA; Department of Biological Sciences, Western Michigan University, Kalamazoo, MI 49008, USA; Department of Biological Sciences, Western Michigan University, Kalamazoo, MI 49008, USA; Department of Biological Sciences, Western Michigan University, Kalamazoo, MI 49008, USA

**Keywords:** enzyme evolution, ancestral sequence resurrection, salicylic acid methyltransferase

## Abstract

We investigated the flowering plant salicylic acid methyl transferase (SAMT) enzyme lineage to understand the evolution of substrate preference change. Previous studies indicated that a single amino acid replacement to the SAMT active site (H150M) was sufficient to change ancestral enzyme substrate preference from benzoic acid to the structurally similar substrate, salicylic acid (SA). Yet, subsequent studies have shown that the H150M function-changing replacement did not likely occur during the historical episode of enzymatic divergence studied. Therefore, we reinvestigated the origin of SA methylation preference here and additionally assessed the extent to which epistasis may act to limit mutational paths. We found that the SAMT lineage of enzymes acquired preference to methylate SA from an ancestor that preferred to methylate benzoic acid as previously reported. In contrast, we found that a different amino acid replacement, Y267Q, was sufficient to change substrate preference with others providing small positive-magnitude epistatic improvements. We show that the kinetic basis for the ancestral enzymatic change in substate preference by Y267Q appears to be due to both a reduced specificity constant, *k*_cat_/*K_M_*, for benzoic acid and an improvement in *K_M_* for SA. Therefore, this lineage of enzymes appears to have had multiple mutational paths available to achieve the same evolutionary divergence. While the reasons remain unclear for why one path was taken, and the other was not, the mutational distance between ancestral and descendant codons may be a factor.

**Significance**

The evolution of protein functions crucial for organismal adaptation occurs by the accumulation of mutations. Here, we show that 2 different mutational paths could have led to the same protein functional change. These findings highlight that while multiple mutational paths may lead to similar solutions for organismal fitness, the reasons why one path is taken over another remain unclear.

## Introduction

It is currently not clear how often multiple different mutational trajectories can promote the evolution of the same novel traits or functions. Experimental evolution and studies of convergence indicate that different lineages often have alternative solutions to evolve the same trait, but this does not indicate that multiple options exist for any singular lineage that may experience contingency (Blount et al. 2018). For instance, intramolecular epistasis is a well-known phenomenon that can limit the number of mutational paths for the origins of novel protein characteristics (Noor et al. 2012; Miton et al. 2021). Whereas positive epistatic substitutions can accumulate in multiple possible orders, if epistasis is negative, evolutionary paths may be restricted such that a particular mutational order is required for adaptive change to occur (Natarajan et al. 2013; Tufts et al. 2015; Starr and Thornton 2016). One powerful approach to help understand how new protein functions arise is to use ancestral sequence resurrection (ASR) (Harms and Thornton 2010). In particular, ancient proteins and their descendants can be studied to identify episodes during which functional changes have occurred (Natarajan et al. 2016; Olson-Manning 2020). This can then be coupled with mutagenesis to identify functionally important changes and their context dependency, if any exists. Such studies have uncovered important evolutionary phenomena related to glucocorticoid receptor ligand binding (Ortlund et al. 2007; Bridgham et al. 2009), hemoglobin adaptation to high elevations (Tufts et al. 2015), and anthocyanin production in flowers (Smith et al. 2013). To understand the potential impact of epistasis on the number of paths available for the evolution of substrate preference switches, we investigated one of the most widely studied plant specialized metabolic enzymes, salicylic acid methyl transferase (SAMT) (Ross et al. 1999).

**Fig. 1.**
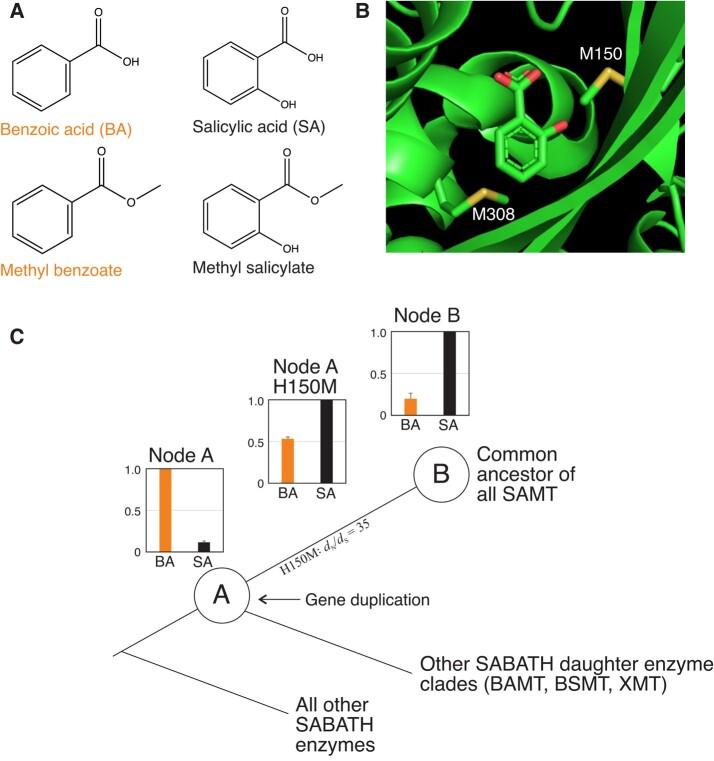
A) Salicylic and benzoic acid differ only in the hydroxyl group on carbon 2 of the phenyl ring. Methylation by SABATH enzymes occurs on the carboxylate moiety. B) The *C. breweri* SAMT crystal structure identified Met150 and Met308 as important residues for SA positioning via a Met-Met clamp (Zubieta et al. 2003). C) Relative enzyme activity assays showed that the ancient SABATH enzyme at node A preferred to methylate BA but after gene duplication the daughter enzyme at node B evolved preference for SA methylation. Statistical analyses suggested that replacement of H150 by Met was positively selected for and experimental mutagenesis demonstrated a role of the residue for substrate discrimination (redrawn from Huang et al. (2012)). Error bars represent standard deviation from assays at each node (Huang et al. 2012). SAMT, salicylic acid MT; BAMT, benzoic acid MT; BSMT, benzoic/salicylic acid MT; XMT, xanthine alkaloid MT.

SAMT is a member of the S-adenosylmethionine (SAM)-dependent SABATH methyltransferase family which is important for the production of many plant specialized metabolites (D'Auria et al. 2003). The SABATH family includes 25 to 40 proteins in most angiosperm species with enzymatic activity against structurally diverse small molecules (Chen et al. 2003; Yang et al. 2006). Several SABATH enzymes have been characterized including those that methylate the carboxyl group of important hormones like indole-3-acetic acid (Zhao et al. 2008) and gibberellins (Varbanova et al. 2007), defensive signaling molecules like jasmonic acid (Seo et al. 2001), and floral fragrance precursors such as benzoic acid (BA; see Fig. 1A) (Pott et al. 2004; Effmert et al. 2005). Methylation of ring nitrogen atoms of stimulants like theobromine is also achieved by SABATH enzymes to produce caffeine (Kato et al. 1999; Uefuji et al. 2003; Huang et al. 2016). SAMT primarily methylates the carboxylate of salicylic acid (SA) to form the product, methyl salicylate (oil of wintergreen) (Fig. 1A), which is an important component of floral scent and also appears to be a mobile signaling molecule for systemic acquired resistance for pathogen defense (Ross et al. 1999; Park et al. 2007; Gong et al. 2023). Because SAMT appears to be involved in the production of methyl salicylate for fundamental signaling processes, its origin and maintenance over time is predicted to be selectively advantageous. Consistent with this prediction is the recent report of conserved SAMT enzyme preference for SA, over the structurally similar BA (Fig. 1A), throughout angiosperm history across nearly every lineage including 41 families (Dubs et al. 2022). A crystal structure for *Clarkia breweri* SAMT was determined in 2003 (Zubieta et al. 2003) and revealed the importance of several residues for SA positioning for methylation in the active site including a Met150–Met308 “clamp” (Fig. 1B). Consistent with the importance of the Met–Met clamp, site-directed mutagenesis of Met150 to the ancestral residue, His, in *Datura wrightii* reduced preference for SA (Barkman et al. 2007).

The origin of SA methylation preference in angiosperms has been previously studied in a historical context using ASR to resurrect the ancestral enzymes that gave rise to the SAMT lineage of proteins (Fig. 1C; Huang et al. 2012). SAMT enzymes evolved from a progenitor that exhibited methylation preference for the structurally similar substrate, BA (Fig. 1C, Node A). After gene duplication and divergence ca. 150 mya, the ability to discriminate SA evolved (Fig. 1C, Node B; Huang et al. 2012). Mutagenesis coupled with enzymatic assays demonstrated that replacement of one amino acid residue in the active site, His150 to Met150 (alignment position 201 in their study), was sufficient to recapitulate the origins of SA substrate preference from the BA-preferring ancestor (Fig. 1C; Huang et al. 2012). This active site residue was reported to have experienced a historical episode of positive selection for replacement (Fig. 1C; Barkman et al. 2007; Huang et al. 2012), presumably to form the Met–Met clamp as shown in the modern-day SAMT crystal structure (Zubieta et al. 2003). No fewer than 5 published studies have used mutagenesis in modern-day and ancestral proteins to show the importance of Met150 for SA preference in different genetic backgrounds (Zubieta et al. 2003; Barkman et al. 2007; Kapteyn et al. 2007; Huang et al. 2012; Han et al. 2018). Yet, recently it has been shown that M150 did not evolve until much later in angiosperm history and was not likely concomitant with the origin of SA methylation preference in this clade (Dubs et al. 2022). Huang et al. (2012) appear to have been misled by the small number of SAMT sequences available for study at that time, all of which had Met150; it is clear now that those lineages independently gained Met150 by convergence no less than 4 times in angiosperm history (Dubs et al. 2022). Thus, it remains unclear what the mutational/structural basis for the evolution of SA preference is in this lineage of conserved enzymes.

To investigate the origin of SA substrate preference in SABATH family enzymes, we resurrected the ancient progenitor of SAMT enzymes and performed mutagenesis. To do this, we assembled a dataset that expands sampling ca. 40-fold over the 2012 study by Huang et al. ASR revealed that, as shown in 2012, the ancient, preduplication ancestor of SAMT preferred to methylate BA over SA. After duplication, the descendant enzyme, from which all angiosperm SAMT have descended, evolved to prefer SA over BA, also as shown in the 2012 study. To recapitulate the substrate preference switch, we investigated the impact of mutations at 4 sites and all possible combinations thereof. Our analyses reveal that a single site was sufficient to switch substrate preference but several positive epistatically interacting sites contributed to an increased level of SA preference over BA. Nonetheless, even in this ancestral enzyme we show that, although the H150M mutation did not occur during this episode of enzymatic divergence, it is sufficient for a complete substrate preference switch. Why this mutational path was not taken remains unclear because there do not appear to be permissive mutations required for its enzymatic effects.

## Results and Discussion

### ASR Reveals that the SAMT Clade Evolved to Prefer SA From an Ancestor that Preferentially Methylated BA

**Fig. 2.**
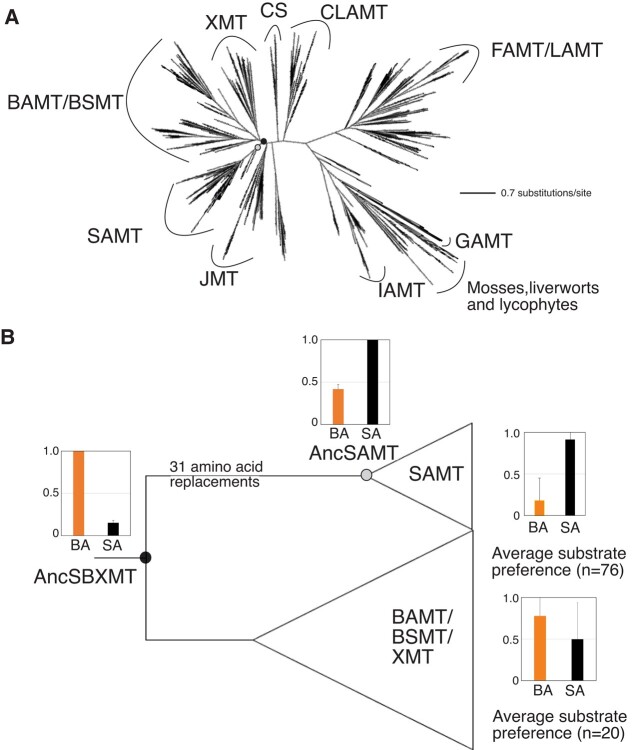
A) Phylogenetic analysis reveals that the SABATH family of methyltransferases is composed of several clades of functionally distinct enzyme lineages. Tree redrawn from Dubs et al. (2022). The node marked by a black dot is AncSBXMT which is the ancestral enzyme that gave rise to all SAMT, BAMT, BSMT, and XMT-type SABATH enzymes. The grey dot marks AncSAMT which is the ancestor that gave rise to all SAMT-type enzymes. B) AncSBXMT is the ancestral enzyme that gave rise to all SAMT, BAMT, BSMT, and XMT-type SABATH enzymes and it prefers to methylate BA relative to SA. After gene duplication, substrate preference switched such that AncSAMT prefers to methylate SA over BA, and this was maintained in most modern-day SAMT. Bars show average and standard deviation. GAMT, gibberellic acid MT; IAMT, indole acetic acid MT; FAMT, farnesoic acid MT; CS, caffeine synthase; CLAMT, carlactonoic acid MT; JMT, jasmonic acid MT; LAMT, loganic acid MT; SAMT, salicylic acid MT; BAMT, benzoic acid MT; BSMT, benzoic/salicylic acid MT; XMT, xanthine alkaloid MT.

Our estimate of SABATH enzyme family history using >1,500 sequences shows that several clades have evolved that appear to exhibit conserved substrate preferences such as JMT, IAMT, and FAMT, although in most cases only one or a few sequences within each have been experimentally characterized (Fig. 2A, supplementary fig. S1, Supplementary Material online; adapted from Dubs et al. (2022)). Within the enzyme family, the SAMT clade is most closely related to BAMT- and XMT-type enzymes (Dubs et al. 2022). In order to determine the origins of distinct substrate preferences shown by modern-day SAMT, we estimated the ancestral sequence for AncSBXMT which gave rise to the SAMT, BAMT, and XMT clades as well as AncSAMT from which all SAMT were derived (Fig. 2B; supplementary fig. S1, Supplementary Material online). These ancestral enzymes are comparable to Nodes A and B from Huang et al. (2012) (Fig. 1C) and statistical confidence in ancestral amino acid positions is high for both nodes (PP = 0.94 to 0.95) (supplementary fig. S2, Supplementary Material online). Our enzyme assays of these ancient proteins revealed that the AncSBXMT progenitor preferred to methylate BA over SA while its descendant, AncSAMT, preferred SA over BA (Fig. 2B). The change in substrate preference shown between AncSBXMT and AncSAMT appears to be one that was largely maintained over the history of the lineage because most SAMT-clade enzymes continue to prefer to methylate SA (Fig. 2B; Dubs et al. 2022). It should be noted that while the majority of BSMT/XMT enzymes prefer to methylate BA (or xanthine alkaloids in the case of many XMT-type enzymes), some prefer to methylate SA, which is the reason for the large variance shown in Fig. 2B. As noted in Dubs et al. (2022), this appears to be due to the fact that those enzymes have compensated for the loss of SAMT-type enzymes in both the Rosaceae and monocots. Our confidence in the substrate preference switch between AncSBXMT & AncSAMT is high because the Huang et al. (2012) study showed the same evolutionary change (see Fig. 1C) even though their ancestral proteins were estimated from far fewer sequences. While the substrate preferences are similar in both studies, the preduplication enzymes are only 83% identical and the postduplication enzymes are only 75% identical which indicates that different portions of the potential ancestral sequence space has been sampled. Therefore, in spite of Huang et al. (2012) being misled in terms of which amino acid positions were replaced between these 2 nodes, inference about change in enzyme activity does not appear to be biased. Since enzyme substrate preference shifted between AncSBXMT and AncSAMT, one or more amino acid replacements other than at the position that is homologous to His150 (Fig. 1B) must explain this change.

### A Significant Relationship Exists Between Residue Distance From Enzyme Active Site and Evolutionary Conservation of Ancient Amino Acid Replacements

**Fig. 3.**
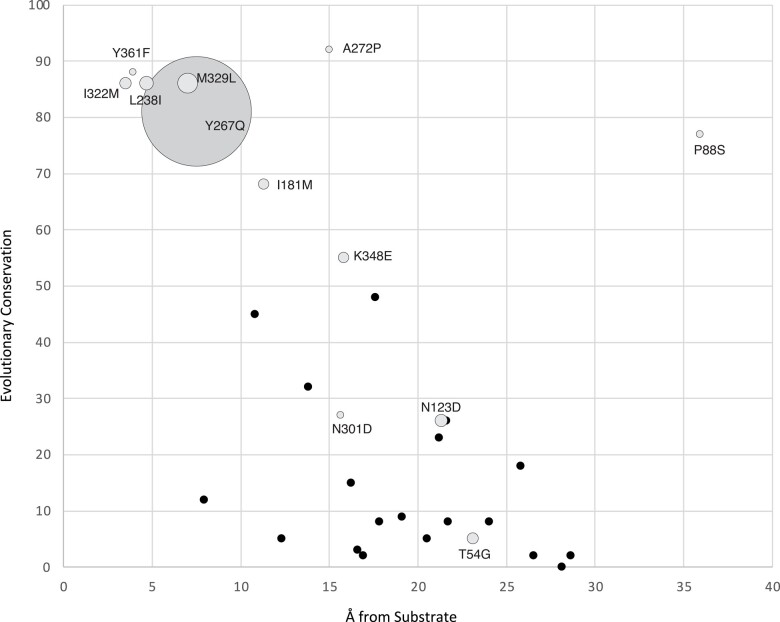
Estimated amino acid residue distance from the substrate in the SAMT enzyme active site is negatively correlated with evolutionary conservation for the 31 positions in AncSBXMT that were replaced by other residues during the divergence of AncSAMT while it acquired SA methylation preference. Solid black circles show amino acid replacements that were not experimentally mutated in AncSBXMT. Grey circles show the 12 amino acid positions that were mutated and characterized in terms of enzyme substrate preference change. The area of the grey circle is proportional to the increase in relative activity with SA as compared to benzoic acid in the mutant relative to wildtype AncSBXMT.

During divergence from its progenitor, AncSBXMT, 31 amino acid replacements appear to have accrued in AncSAMT (Fig. 2B; supplementary fig. S2, Supplementary Material online). In order to understand which of these replacements may have been important for the evolution of SA preference, we determined the relationship between residue proximity to the active site and its degree of evolutionary conservation (Fig. 3). Our sampling included 66 functionally characterized angiosperm SAMT-type sequences representing every major lineage as found in Dubs et al. (2022). A statistically significant relationship exists such that those sites that are highly conserved in modern-day angiosperm SAMT enzymes are found nearer to the position of SA as bound in the enzyme active site of *C. breweri* (*r* = −0.54; *P* < 0.05; Fig. 3). This conclusion appears robust to differing methods of measurement of the 2 parameters (*r* = 0.46; *P* < 0.05; supplementary fig. S3, Supplementary Material online). Only 11 residues are <15 Å from the substrate in the active site and, of those, only 6 are conserved in more than 50% of the characterized SAMT enzymes (Fig. 3). The fact that these 6 sites are close to the substrate binding pocket (<10 Å) and highly conserved in nearly all 66 SAMT enzymes descended from AncSAMT, strongly suggests that they were important for the evolution of SA preference over BA and have likely remained so.

### The Largest Functional Effects are due to Historical Mutations to Residues that are Close to the Active Site and Highly Conserved

To study the effects of a subset of the 31 amino acid replacements that are inferred to have occurred between AncSBXMT and AncSAMT, we made 12 single-site mutations that span the range of evolutionary conservation and distance from the active site in order to determine if residues that are more conserved and closer to the substrate have larger effects on enzyme activity (Fig. 3). Enzyme assays of the 12 mutants show that Y267Q exhibited the largest increase in SA preference relative to wildtype (Fig. 3). The other positions had much smaller effects on SA preference with M329L having the second highest impact. The average increase in SA-preference is higher if the mutated sites are <10 Å from the substrate and more highly conserved; however, this is largely due to the impact Y267Q (Fig. 3). We did not intend to have a complete characterization and statistical analysis of all possible mutants; therefore, we cannot exclude potentially important roles for the other inferred substitutions. I322M was predicted to be involved in the substrate preference switch since it forms part of the modern-day Met–Met clamp (Zubieta et al. 2003; Fig. 1B) but its functional effect is minimal when introduced alone (Fig. 3). The 2 curious outlier mutations, P88S and A272P, were more highly conserved than others that were similarly distant from the active site; yet, their impact on a substrate preference switch towards SA was minimal. Therefore, it remains unclear why they are so highly conserved amongst modern-day SAMT enzymes in spite of being far from the bound substrate.

Of the 12 sites that we mutated in AncSBXMT, 9 were also inferred to have changed between Node A and B in the 2012 study by Huang et al. (Fig. 1C). In order to verify our conclusions about the functional importance of the single-site mutants we generated here, we made 3 mutations to the ancestor of Node A from Huang et al. (2012) (Fig. 1C). Consistent with the results obtained for AncSBXMT mutants, the Node A Y267Q mutation also had the largest effect on SA preference (supplementary fig. S4, Supplementary Material online).

### One Historical Mutation Alone Switched Substrate Preference but Small Positive Epistatic Effects Appear to Amplify its Magnitude

**Fig. 4.**
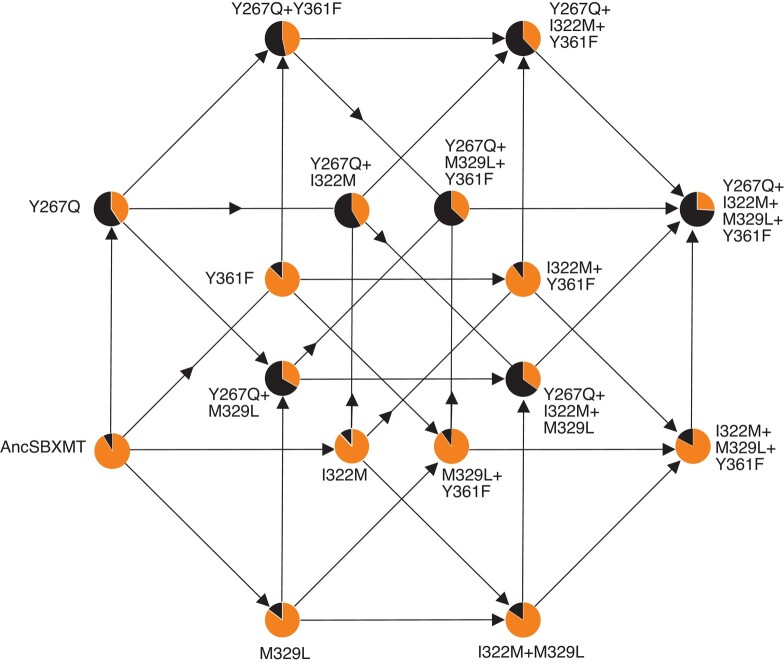
The origin of AncSAMT preference for SA methylation likely originated with Y267Q in AncSBXMT. Circles are colored according to the relative enzyme activity with BA and SA. Our enzymatic characterizations of the 15 single, double, triple, and quadruple mutants show that only when site Y267 is replaced by Q is enzymatic preference for SA relative to BA switched. However, the other 3 amino acid replacements appear to be positively epistatic. There are 24 possible evolutionary paths for 4 mutations of AncSBXMT and all possible combinations thereof.

**Fig. 5.**
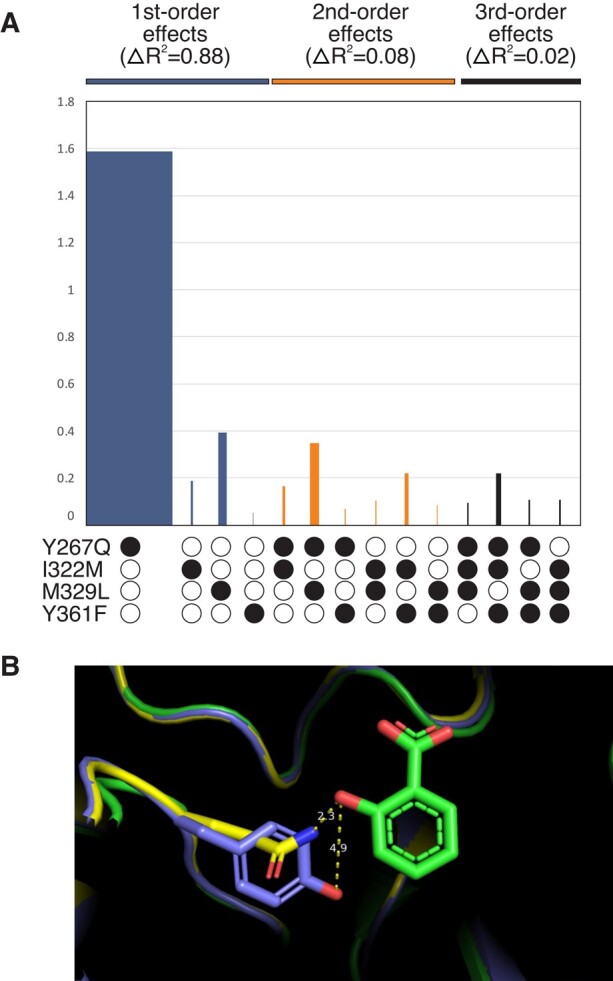
A) Linear regression modeling estimates of first-, second- and third-order effects of mutations on enzyme preference for SA relative to benzoic acid. Height of bars shows estimated effect coefficients from the best-fit model and width of bars illustrates *R*^2^ of each mutation or combination thereof. Y267Q has the largest effect on substrate preference and explains 80% of the variation observed. B) AlphaFold model of AncSBXMT and the AncSBXMT Y267Q mutant aligned to *C. breweri* SAMT (1M6E). Although the models are very similar, the position of the amide of Q267 in the mutant is predicted to be favorably positioned for hydrogen bonding with SA at 2.3 Å whereas the hydroxyl of the phenyl ring of Y267 in AncSBXMT is not.

To investigate if epistatic interactions exist amongst 4 of the most evolutionarily conserved active site residues that were replaced during the divergence of AncSAMT, we made all possible single, double, triple, and quadruple mutants and functionally characterized them in terms of substrate preference. The 4 mutated sites, Y267Q, I322M, M329L, and Y361F, are all within 10 Å of active site and conserved in >80% of modern-day SAMT descendants and each has a high posterior probability of having been replaced during the interval of enzymatic divergence (Fig. 3; supplementary figs. S2 and S5, Supplementary Material online). As shown in Fig. 4, any of the 15 mutants that possessed Y267Q had the highest ability to discriminate SA from BA. And, notably, no combination of mutations lacking Y267Q preferred to methylate SA over BA (Fig. 4; supplementary table S1, Supplementary Material online). The other single mutants, I322M, M329L, and Y361F, provided only minimal improvement of SA methylation relative to BA. When adding together the increases in SA preference from each single mutant, the predicted combination would not provide the level of substrate discrimination seen in the quadruple mutant (supplementary table S1, Supplementary Material online). Specifically, the quadruple mutant showed a 2.8-fold preference for SA whereas if the 4 mutations are strictly additive, only a 1.9-fold preference for SA would be predicted (Fig. 4). Therefore, we hypothesized that positive-magnitude epistatic interactions must exist between the residues. When we used multiple linear regression modeling (Miton et al. 2020), a model which included second- and third-order interaction terms was a significantly better fit than the no epistasis or second-order interaction model (*P* < 0.05). Figure 5A shows the estimated effect coefficients as well as the *R*^2^ associated with each mutation or combination thereof in the best-fit model. While the impact of Y267Q alone is obvious, there are small positive interaction effects when any of the other sites are in combination, especially that of Y267Q+M329L. In contrast to other studies, we found no evidence for negative intramolecular epistatic interactions (Smith et al. 2013; Miton et al. 2020).

The relative impacts of selection and genetic drift on the fixation probability of a particular allele depend on ancestral population size and the strength of fitness effects due to the mutation. Assuming that Y267Q would provide an immediate selective advantage due to more efficient methyl salicylate production in ancestral angiosperms (Fig. 4), its persistence would be enhanced more so than any other mutation investigated in this study. Therefore, it is most probable that the mutational path to AncSAMT originated via Y267Q because none of the other 11 mutations alone would provide a catalytic advantage in terms of improving SA methylation (Fig. 3). Afterwards, though, any of the other 3 mutations could accumulate in any order without significantly reducing activity with SA (Fig. 4). It remains unclear if any of the other 27 mutations that accumulated would show negative epistatic interactions with Y267Q.

To investigate the kinetic basis for the substrate preference shift shown, we estimated apparent Michaelis–Menten parameters for wild type AncSBXMT and the Y267Q mutant with each of the 2 substrates, SA and BA. Table 1 shows that the relative *k*_cat_/*K_M_* estimated in these experiments are consistent with our competitive substrate assays in that AncSBXMT prefers BA over SA by more than 10-fold while the Y267Q mutant prefers SA over BA slightly more than 1-fold. Evaluation of separate apparent *K_M_* and *k*_cat_ parameter estimates reveals that the decreased relative preference for BA in Y267Q appears to be due to both a decrease in *k*_cat_ and increase of *K_M_*. On the other hand, the increased relative preference for SA methylation is largely due to a nearly 4-fold decrease in *K_M_*. Since the *K_M_* for SA is improved in the Y267Q mutant, we predict that the Y267Q replacement might enhance binding of SA in some way.

**Table 1:** Apparent enzyme kinetic parameter estimates for AncSBXMT and AncSBXMT Y267Q with 2 competing substrates. Standard error is shown for each parameter estimate (*n* = 2)

Enzyme (substrate)	*K_M_* (μM)	*k*_cat_ (1/s)	*k*_cat_/*K_M_* (s^−1^M^−1^)
AncSBXMT (BA)	961 ± 260	0.0029 ± 0.00027	2.96
AncSBXMT (SA)	7596 ± 2096	0.0007 ± 0.00013	0.09
AncSBXMT Y267Q (BA)	1938 ± 202	0.0007 ± 0.00005	0.37
AncSBXMT Y267Q (SA)	1955 ± 299	0.0008 ± 0.00007	0.43

### Computational Prediction of Structural Basis for Substrate Preference Evolutionary Shift

In order to visualize the positioning of the ancient protein side chains, we used AlphaFold (Jumper et al. 2021) to generate predicted models of AncSBXMT and the associated Y267Q mutant. These models were aligned to SAMT from *C. breweri* (Zubieta et al. 2003) to determine if Y267Q might alter the active site architecture to explain the evolution of SA preference. The AlphaFold model for AncSBXMT predicts that the side chain of Y267 would be angled away from SA in the active site by 4.9 Å (Fig. 5B). In contrast, in the AncSBXMT Y267Q model, the sidechain of Q is predicted to be angled 90° towards SA and is in a favorable position for H-bonding to the 2-hydroxyl of the substrate at ca. 2 Å. This alteration could explain the decreased *K_M_* for SA due to hydrogen bonding in AncSBXMT Y267Q (Fig. 5B). Because these inferences are based solely on computational modeling, other explanations could be rationalized; since Y267 is longer than Q it is possible that AncSBXMT does not prefer to methylate the larger substrate, SA, due to steric hindrance. Future studies that crystalize the AncSBXMT and Y267Q mutant proteins, with each of the 2 substrates bound, could reveal the basis for the substate preference change we have documented.

### H150(157)M: The Mutational Path not Taken

Huang et al. (2012) previously reported H150M (Fig. 1B) switched enzyme substrate preference from BA to SA in their Node A ancestral enzyme (Fig. 1C). In order to test the hypothesis that this mutation would also switch substrate preference in the comparable AncSBXMT background, we replaced H157 with Met, which is the homologous position to M150 in *C. breweri* SAMT (Fig. 1B). This mutation alone switched substrate preference to SA from BA and the magnitude of its effect is much greater than any other single mutation we investigated in this study (supplementary fig. S4, Supplementary Material online). Thus, the Huang et al. (2012) study was not misled about the effect of this mutation; however, although it represents an alternative evolutionary solution for the origin of SA preference, it was a mutational path not taken in the SAMT lineage of enzymes (*P* < 0.05; supplementary fig. S2A, Supplementary Material online). One reason this path was not followed might be that the codons for His and Met differ at all 3 positions. Therefore, unless an amino acid residue that is encoded by a codon that is intermediate between His and Met provided an advantage (or is at least neutral), the path might be closed since it would require 3 contiguous and simultaneous mutations. Multinucleotide mutation is a process that could enhance the probability of this replacement, though (Schrider et al. 2011). It is also possible, but perhaps less likely, that if the ancestral CAU codon for His was inverted, it would result in a change to AUG with a single mutational step and would encode for Met. However, we are unaware of studies showing inversions occurring to such short segments of a chromosome. Furthermore, since mutation is a stochastic process, it is also possible that the H157M replacement did not occur during the origin of AncSAMT simply due to chance alone. A different possible explanation for why the H157M mutational path was not followed could be that it results in reduced protein stability or somehow otherwise compromises enzyme activity such that it would not be ancestrally favored. Whatever the reason this evolutionary path was not taken during the origin of SA preference in this lineage of enzymes, Met has evolved in this homologous position several times in the history of SABATH enzymes, in spite of the large mutational distance. In fact, as previously reported, phylogenetic analysis of variance (ANOVA) shows the correlation of Met 150 with SA preference over BA in angiosperm SABATH enzymes including SAMT, BAMT, and XMT (Fig. 2A) (Dubs et al. 2022). An analysis of ancestral states by Dubs et al. (2022) suggests that in SAMT, His was first replaced by Gln which then later evolved to Met at least 3 times. Given that Gln is potentially only 2 mutations from Met, this may represent an intermediate that maintains SA preference.

While we have demonstrated that more than one mutational path could have resulted in similar evolutionary shifts in the origin of SAMT-type enzymes in angiosperms, it is unknown whether additional mutational paths could also have been taken. High-throughput methods for functional assays coupled with saturation mutagenesis allow such questions to be rigorously investigated in certain systems (Starr et al. 2017; Park et al. 2022). For other enzyme systems without high-throughput assays, like SAMT, reliance upon explicitly historical approaches like the one we have used here may be necessary. However, as we show, such historical studies need to be cognizant of the potential impact convergent evolution can have on statistical inferences. In the case of SAMT, the combination of repeated convergent evolution of the H150M replacement and insufficient sequence sampling led to the fortuitous, but misleading, discovery of a mutational path not taken. While the genomic/transcriptomic era should alleviate the impact of insufficient sampling for future studies, the fact that statistical approaches to estimate ancestral sequences and positive selection can be misled in the face of convergence, especially if multinucleotide mutations have occurred (Venkat et al. 2018), should signal caution for evolutionary studies more broadly.

## Materials and Methods

### Ancestral Sequence Resurrection

The tree shown in Fig. 2A and supplementary fig. S1, Supplementary Material online was modified from Dubs et al. (2022). Although the methods for sequence sampling and tree estimation have already been described there, we provide a synopsis here since ASR was performed using those data. Using the *C. breweri* SAMT (AF133053.1) as a query sequence, transcriptome and genome amino acid sequences were obtained by performing a BLAST search of sequence data available in the Phytozome, OneKP, and Genbank databases. Additional sequences were obtained using BLAST searches of the *Azolla filiculoides* and *Salvinia cucullata* (Li et al. 2018), *Ginkgo* (Guan et al. 2016) and *Picea abies* (Nystedt et al. 2013) genomes. Sequences were aligned using MAFFT version 7 (Katoh and Standley 2013) using the auto strategy and default parameters. From the alignment, Maximum likelihood was used to estimate a tree as implemented in IQTree (Nguyen et al. 2015). Trees were generated assuming a Jones–Taylor–Thornton model of amino acid substitution assuming a number of FreeRate categories as determined by Model Finder (Kalyaanamoorthy et al. 2017). Bootstrap estimates were generated using IQTree's ultrafast bootstrapping method (Hoang et al. 2018) on the CIPRES platform. Assuming the phylogenetic tree obtained from the 1,500 sequence dataset, the AncBSXMT and AncSAMT progenitor protein estimates (Fig. 2 and supplementary fig. S1 and S2, Supplementary Material online) were obtained from IQTree using the Empirical Bayesian approach. To estimate ancestral amino acid presence or absence in length variable regions, we generated a second alignment file in which all amino acids in the original alignment were replaced with a “1” and all gaps in the alignment were replaced with a “0”. With this binary file, we used IQTree to estimate the likelihood of an amino acid being present in all positions for each node. The chosen model for the binary dataset was: GTR2 + FO + ASC + R10. This is a general time reversible model for binary data (GTR2) with 10 free rate categories (R10). The FO option optimizes state frequencies by maximum likelihood and ASC provides ascertainment bias correction. Supplementary fig. S2, Supplementary Material online shows an alignment of the 2 estimated sequences that were synthesized and experimentally characterized.

### Synthesis and Cloning of Ancestral Sequences

Ancestral sequence estimates were submitted to GenScript Corp. (Piscataway, NJ, USA) for synthesis and codon optimization for expression in *Escherichia coli* cells. Sequences were also designed to avoid matching BamHI and NdeI restriction sites which were later used for subcloning. The synthesized genes were excised from the pUC57 cloning vector using BamHI and NdeI restriction enzymes. Restriction products were isolated from the reaction mixture via gel electrophoresis and purification using the Promega Wizard SV Gel and PCR Clean-Up System kit. After purification, ancestral sequences were subcloned into the pET15b (Novagen) expression vector and subsequently transformed into XL-10 gold super-competent *E. coli* cells. Transformed cells were plated onto LB agar containing 100 μg/mL ampicillin and incubated overnight at 37 °C. Colonies from the plates were used to inoculate 5 mL LB starter cultures selected with 100 μg/mL ampicillin and incubated overnight at 37 °C. Starter cultures were then used to prepare minipreps using the Promega Wizard Plus SV Minipreps DNA Purification System kit. Electrophoresis using a 1.5% agarose gel was used to screen for full-length plasmid inserts which were sent to Genewiz (AZENTA Life Sciences, South Plainfield, NJ) for Sanger sequencing. Error-free plasmids were transformed into BL21 Star (DE3) OneShot Chemically Competent Cells for protein expression onto LB plates containing 100 μg/mL ampicillin following the manufacturer's protocol (Invitrogen Corp., Carlsbad, CA).

### Site-directed Mutagenesis

Mutagenesis was carried out using the Agilent QuikChange Lightning Kit (Agilent Technologies Inc., Santa Clara, CA) following the manufacturer's protocol. Primers (supplementary table S2, Supplementary Material online) were designed using the Agilent QuikChange Primer Design program. Polymerase chain reactions (PCR) were carried out as follows: initial denaturation at 95°C for 2 min, followed by 18 cycles of denaturation at 95°C for 20 s, annealing at 60°C for 10 s, and final elongation at 68°C for 3.5 min followed by an additional 68°C final extension for 5 min. Following Dpn1 digestion, PCR products were transformed into XL10-Gold Ultracompetent *E. coli* cells. One hundred microliters of transformed cells were plated onto LB plates containing 100 μg/mL ampicillin. Transformed colonies were then grown overnight in 5 mL LB cultures containing 100 μg/mL ampicillin at 37°C. Miniprep purification and sequencing was performed as described above. After the desired mutation(s) were confirmed, plasmids were transformed into BL21.

### Relative Enzyme Assays

Relative substrate preferences of ancestral proteins and single-site mutants shown in Fig. 3 were determined using a previously designed competitive assay that was run in 50 mL *E. coli* cultures (Ross et al. 1999; Barkman et al. 2007; Dubs et al. 2022). Overnight cultures of transformed BL21 cells were grown at 37°C in 5 mL LB media containing 100 μg/mL ampicillin. Cultures were scaled up to 50 mL LB with ampicillin selection the next day and incubated at 37°C at 225 rpm until an optical density of ca. 0.6 at 600 nm was reached. Gene expression of pET15b was then induced using a final concentration of 1 mM isopropyl β-d-1-thiogalactopyranoside (IPTG). Competitive assays were initiated by the addition of 1 mM SA and 1 mM benzoic acid directly to the induced cultures that were grown on a shaker at room temperature for 4 h. Cultures were subsequently centrifuged at 4,000 rpm at 4°C for 20 min to pellet cells. The supernatant was recovered and then extracted using 5 mL of hexane. After separation, the upper phase was removed and stored at −20°C until GC-MS analysis occurred.

### Protein Purification and Competitive Substrate Assays

In order to conduct assays of 4 single and 11 higher-order mutants to investigate potential epistatic interactions, 5 mL overnight cultures of transformed BL21 cells were grown at 37°C in LB media containing 100 μg/mL ampicillin. Cultures were scaled up to 50 mL the next day and incubated at 37°C until mid-log phase was reached. After inducing with 1 mM IPTG, the cultures were grown at room temperature for 6 h. Cell pellets were obtained by centrifugation at 4°C and 4,000 rpm for 20 min. Supernatant was discarded and pellets were resuspended in 3.6 mL of chilled EWB buffer (50 mM Na_2_HPO_4_, 300 mM NaCl, 10 mM imidazole, 12% glycerol (v/v), pH 8). Cells were sonicated on ice 3 times for 30 s intervals with 20 s of rest in between. Lysates were then centrifuged at 4°C and 4,000 rpm for 20 min after which the supernatant was decanted and stored at −80°C until the protein was purified. Protein purification was performed using the Takara TALON Spin Column kit (Clontech Laboratories, Inc., Mountain View, CA). Purified protein was mixed with 10% glycerol and 12.5 mM DTT and stored at −80°C until later use. Precast NuPAGE 10% Bis–Tris gels were run to confirm protein sizes and purity. Gels were stained using the SimplyBlue SafeStain microwave protocol (Thermo Fisher Scientific Inc., Waltham, MA). Bradford assays were done to determine protein concentrations using the Coomassie Plus Assay Reagent following the manufacturer's protocol (Thermo Fisher Scientific Inc., Waltham, MA). In order to empirically determine relative substrate preference for SA versus BA in AncSBXMT and single-site mutants shown in Fig. 4, we used 250 µL competitive assays. To each reaction, we added 10 to 40 μg purified protein, 50 mM Tris Buffer (pH 7.5), 0.64 mM nonradioactive SAM, 1 mM SA, 1 mM benzoic acid, and water to bring the final reaction volume to 250 μL. The reactions incubated at room temperature overnight. Reaction products were extracted using 250 μL of hexane by inversion. Once separated, the upper phase was removed and stored at −20°C for GC-MS analysis.

### GC-MS Analysis

One microliter of hexane extracts from all enzyme assays were analyzed using GC-MS on an HP6890 GC system coupled to an HP5973 Mass Selective Detector equipped with a DB-5 capillary column. The inlet was splitless and supplied with He gas with a flow rate of 1 mL/min. Oven conditions included an initial hold at 40°C for 2 min followed by ramping 20°C/min to 300°C followed by a 2 min hold at the final temperature. We scanned for ions in the mass range between 50 and 550 amu. We quantified the relative amounts of methyl benzoate and methyl salicylate in all samples by integrating the area beneath the respective peaks. These areas are directly comparable because the ionization efficiencies for the 2 metabolites are approximately equal as determined by analysis of known concentrations of standards.

### Enzyme Kinetics

To estimate apparent Michaelis–Menten kinetic parameters, enzyme assay conditions were first evaluated to ensure linearity of reaction velocity in terms of purified protein concentration and time with nonsaturating substrate concentrations. Estimates for *K_M_* and *k*_cat_ were then determined by varying methyl acceptor substrate concentration while protein concentration and time were constant. The ^14^C-labeled S-adenosylmethionine methyl donor concentration was also held constant and saturating at 320 µM. Radiochemical assays were performed in 50 µL reactions with 0.01 mCi (0.5 µL) labeled SAM, 1 µL methyl acceptor substrate (BA or SA) and 10 to 20 µL purified protein in 50 mM Tris–HCl buffer at 24°C. Radiolabeled methylated products were extracted with ethyl acetate and counts per minute were obtained using a Perkin–Elmer Tri-Carb 2910 TR Scintillation counter. Kaleidagraph Version 5 (Synergy Software, Reading, PA, USA) was used to fit saturating curves to estimate Michaelis–Menten parameters (supplementary fig. S6, Supplementary Material online).

### Statistical Analysis

We used correlation analysis to determine whether there is a significant relationship between the proximity of a mutation to the active site and its degree of evolutionary conservation. The proximity of 31 amino acid replacements inferred between AncSBXMT and AncSAMT (supplementary fig. S2, Supplementary Material online) to the active site was measured in Angstroms using PyMol (Version 2.0: Schrödinger, LLC). To do this, we identified each position in the *C. breweri* SAMT crystal structure (1M6E) that is homologous to the historical ancestral amino acid replacements. From these, we determined the distance from the residue to the closest point of the substrate within the binding pocket. Evolutionary conservation of each of the 31 inferred replacements was determined by counting the number of modern-day SAMT sequences that retained the amino acid. The dataset used for the modern-day SAMT sequences included the experimentally studied enzymes from Dubs et al. (2022). To investigate any potential bias we performed an additional analysis utilizing different methods for quantifying each of the 2 parameters. First, to use a more uniform method of measuring amino acid position to the substrate, we measured from its alpha-carbon atom to the carbon atom of the carboxyl moiety of SA using PyMol. In order to take phylogeny into account in assessing evolutionary conservation (or lack thereof), we used maximum parsimony in MacClade v. 4.0 (Maddison and Maddison 2001) to quantify the number of subsequent changes that have occurred at a particular alignment position that was replaced along the branch linking AncSBXMT and AncSAMT.

To study whether there are intramolecular epistatic interactions amongst mutations, we used linear regression modeling (Miton et al. 2020) on the measured ratio of the amount of methyl salicylate produced relative to the amount of methyl benzoate produced by each single and higher-order mutant in competitive assays. The computer code for the statistical analysis of epistasis using linear regression was developed by Miton et al. (2020) and proceeds in a stepwise manner. First, the so-called first-order model is generated in which no interactions between mutated sites are allowed. Once the best first-order model and its set of parameters is obtained, it is compared to a more complicated model in which all possible pairwise mutation interactions are included. If the model with pairwise interactions between mutations is a significantly better fit to the data, then the simpler first-order model is rejected, thereby providing evidence for epistatic interactions amongst mutations. These comparisons can be extended to investigate increasingly higher-order interactions (third-order, fourth-order, etc.). In all cases, because simpler models are nested within more complex ones, an F-test is used to compare them. The model comparisons end when a simpler model cannot be rejected in favor of a more complex one with higher-order interactions. In this case, effect coefficients from each combination of positions in the model are extracted from the final model. For a more complete description of the method, readers should refer to Miton et al. (2020).

The ratio of specificity constants, *k*_cat_/*K_M_*, indicates enzyme preference for one substrate relative to another (Fersht 1999). We empirically approximated this ratio for the statistical analysis of epistasis by performing competitive enzyme assays, using equimolar substrate concentrations, as described above. Use of the competitive assays as an approximation for the ratio of apparent specificity constants appears justified based on our limited comparisons. AncSBXMT prefers BA over SA by 11-fold in competitive assays and the preference for BA over SA is 31-fold according to the ratio of *k*_cat_/*K_M_* (Table 1). AncSBXMT prefers SA over BA by 1.3-fold in competitive assays, and it is 1.2-fold according to ratio of *k*_cat_/*K_M_* (Table 1). We see these as comparable measures of substrate preference. Regardless of how well the competitive assays are accurate representations of the ratio of specificity constants, they are the most direct measure of which substrate will be preferred by these enzymes when in equimolar concentration.

## Supplementary Material

evae016_Supplementary_Data

## Data Availability

The data matrix underlying ancestral state estimation reported in this article is available at https://figshare.com/articles/dataset/Figure 2_alignment_for_ASR_fasta/25050875.
