# Supplementary material for: The Mutational Road not Taken: Using Ancestral Sequence Resurrection to Evaluate the Evolution of Plant Enzyme Substrate Preferences
Source: Genome Biol Evol. 2024 Jan 30;16(2):evae016. doi: 10.1093/gbe/evae016 (PMC10853004; doi:10.1093/gbe/evae016)
Supplement: evae016_Supplementary_Data [file evae016_supplementary_data.pdf]

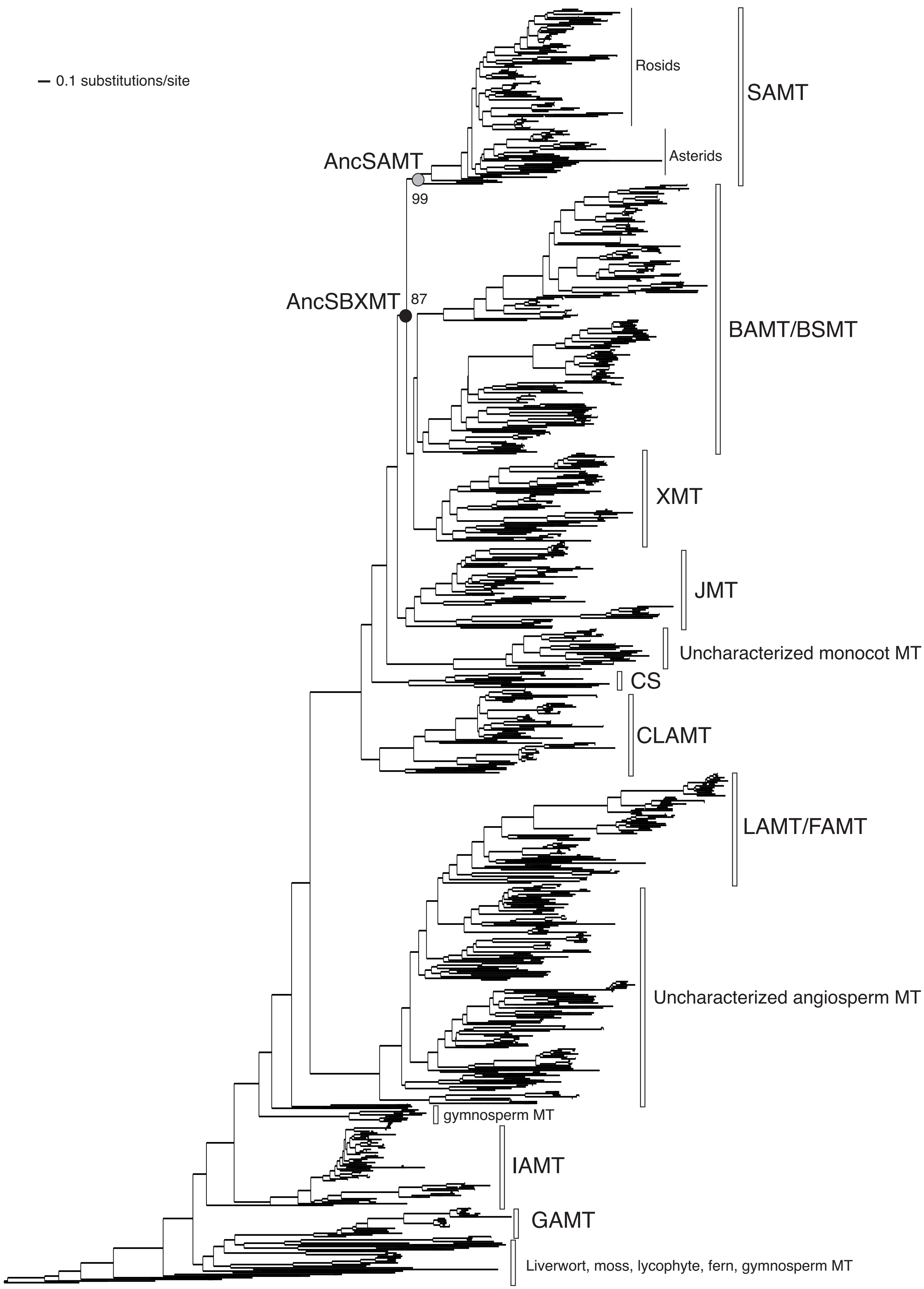

Supplementary Fig. S1. SABATH enzyme family phylogeny from which ancestral sequence estimates were obtained. Bootstrap values are shown for the two nodes representing the ancestral enzymes resurrected for functional analysis.

A.

```
                 10        20        30        40        50        60
AncBSXMT MEVQQVLHMNGGDGETSYANNSSLQKKVISMAKPIIEEAITDLYCSNTFPESLTIADLGC
         ::: ::::::::::::::::::::::::::::::: :::: ::::: :::::: ::::::
AncSAMT  MEVVQVLHMNGGDGETSYANNSSLQKKVISMAKPITEEAILDLYCS-TFPESLGIADLGC
                 10        20        30        40         50       60

                 70        80        90       100       110       120
AncBSXMT SSGPNTLLVVSEIIDTIHKKCRQLGRPPPEFQVFLNDLPGNDFNTIFRSLPSFYEKLKKE
         ::::::::::::::::::::::::::: ::::::::::::::::::::::::::::::.
AncSAMT  SSGPNTLLVVSEIIDTIHKKCRQLGRPSPEFQVFLNDLPGNDFNTIFRSLPSFYEKLKEG
                 70        80        90       100       110       120

                                          H157: 0.99
                130       140       150       160       170       180
AncBSXMT KGNGFGPCFIAGVPGSFYGRLFPSKSLHFVHSSYSLHWLSQVPQGLENEAGGVLNKGNIY
         .:. :::::::::::::::::::::::::::::::::::::::::::.:::. .::::::
AncSAMT  NGDDFGPCFIAGVPGSFYGRLFPSKSLHFVHSSYSLHWLSQVPQGLESEAGSPINKGNIY
                130       140       150       160       170       180
                                          H157: 0.99

                190       200       210       220       230       240
AncBSXMT IAKTSPPSVLKAYLEQFQRDFSLFLKSRSEEMVPGGRMVLTLLGRRSADPSSKECCYLWE
         .::::::::::::::::::::::::::::::::::::::::::::::::::::::::.::
AncSAMT  MAKTSPPSVLKAYLEQFQRDFSLFLKSRSEEMVPGGRMVLTLLGRRSADPSSKECCYIWE
                190       200       210       220       230       240
                              Y267:
                250       260 0.97  270       280       290       300
AncBSXMT LLAQALNDMVSEGLIEEEKLDSFNLPYYAPSAEEVKAVIEKEGSFTIDRLETFEVNWDAN
         :::::::::::::::::::::::::: :::: ::::.:::::::::::::::::::::..
AncSAMT  LLAQALNDMVSEGLIEEEKLDSFNLPQYAPSPEEVKSVIEKEGSFTIDRLETFEVNWDSE
                250       260 Q267: 270       280       290       300
                    I322: 0.97 0.70  M329: 0.91
                310       320       330       340       350       360
AncBSXMT NNDDDGNKSDKLKRGKNVAKYIRAVAEPMLASHFGEAIMDELFRRYAKIVAEHMSKEKTK
         ..  :.   ::::::::::::.::::::.::::::::::::::::: .::::::::::::
AncSAMT  DDPGDNLAFDKLKRGKNVAKYMRAVAEPLLASHFGEAIMDELFRRYREIVAEHMSKEKTK
                310       320       330       340       350       360
                   M322: 0.85        L329: 0.97
  Y361: 0.76    370
AncBSXMT YINLVISLTKK
         .::::::::::
AncSAMT  FINLVISLTKK
                370
  F361: 0.70
```

B.

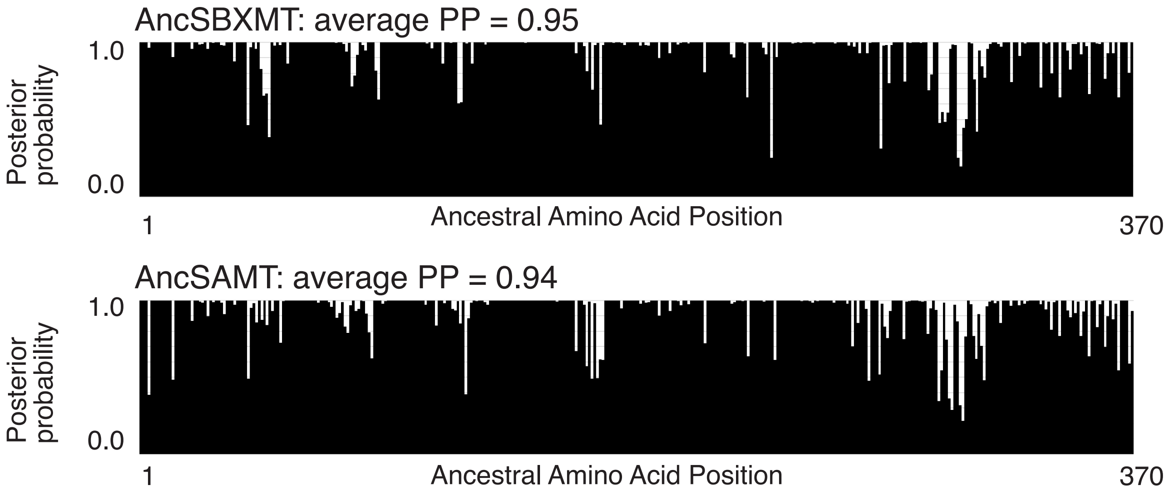

Supplementary Figure S2. Comparisons of estimated ancestral SABATH enzymes that gave rise to modern-day SAMT enzymes in angiosperms. A. Alignment of AncSBXMT and AncSAMT. Positions within green boxes are those sites that were mutated in order to investigate any potential epistasis amongst the 4 sites that were replaced during the origin of SA-methylation preference by AncSAMT. B. Site-specific posterior probabilities for each position within the resurrected proteins.

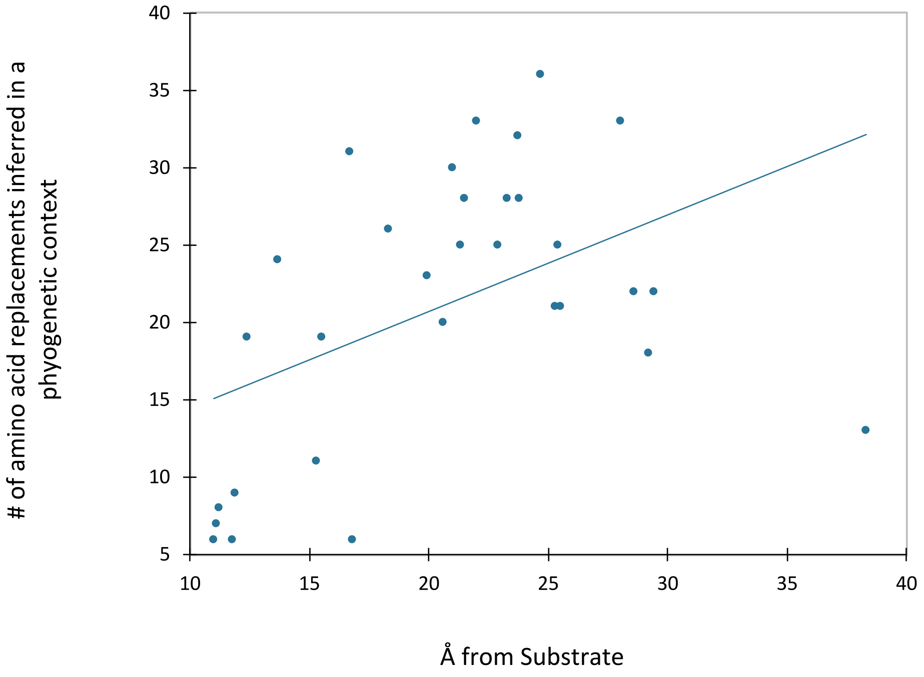

Supplementary Figure S3. Regression analysis of the distance from ancestral amino acid replacements at particular positions to the substrate as bound in the active site of SAMT and the inferred numbers of changes at those positions after their evolutionary origins. Distance to the substrate was measured from the alpha-carbon atom of each amino acid to the carbon atom of the carboxyl group of the substrate, salicylic acid. The number of inferred changes at each amino acid position was estimated using parsimony on the phylogenetic tree showing relatedness amongst the enzymes (Dubs et al., 2022)

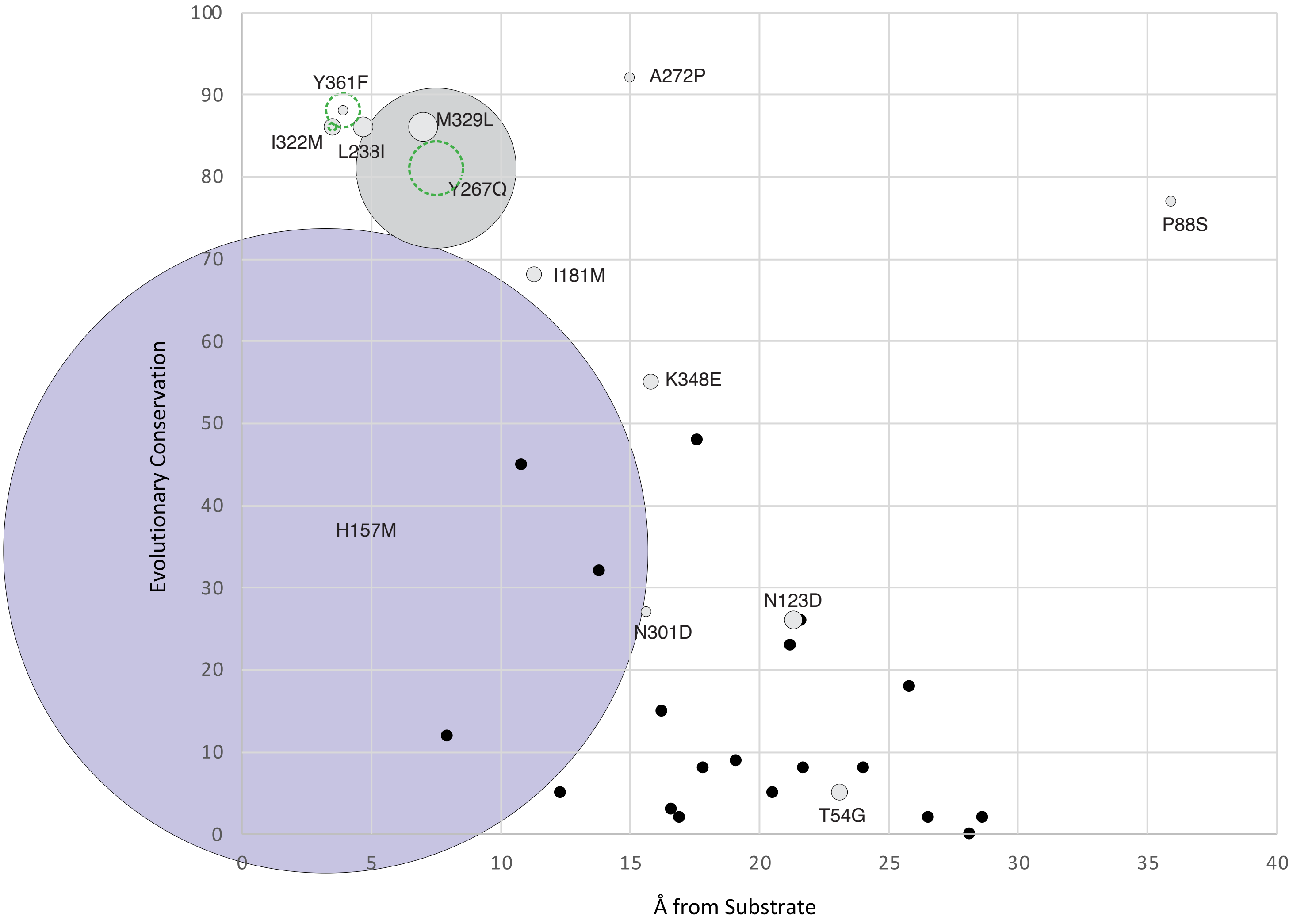

Supplementary Figure S4. Amino acid residue distance from the substrate in the enzyme active site is negatively correlated with evolutionary conservation for the 31 positions that were replaced in AncSBXMT as AncSAMT diverged and acquired SA methylation preference. Solid black circles show amino acid replacements that were not experimentally mutated in AncSBXMT. Grey circles show the 12 amino acid positions that were mutated and characterized in terms of enzyme substrate preference change. The area of the grey circle is proportional to the increase in relative activity with salicylic acid as compared to benzoic acid in the mutant relative to AncSBXMT. The green dotted circles show the impact of the corresponding mutation in the Node A background from the Huang et al., 2012 study. While H157M is not predicted to have occured during the divergence of AncSBXMT towards AncSAMT, we plotted its position on this chart for comparative purposes and show the improvement with SA methylation as a mauve circle proportion to its increase over wildtype.

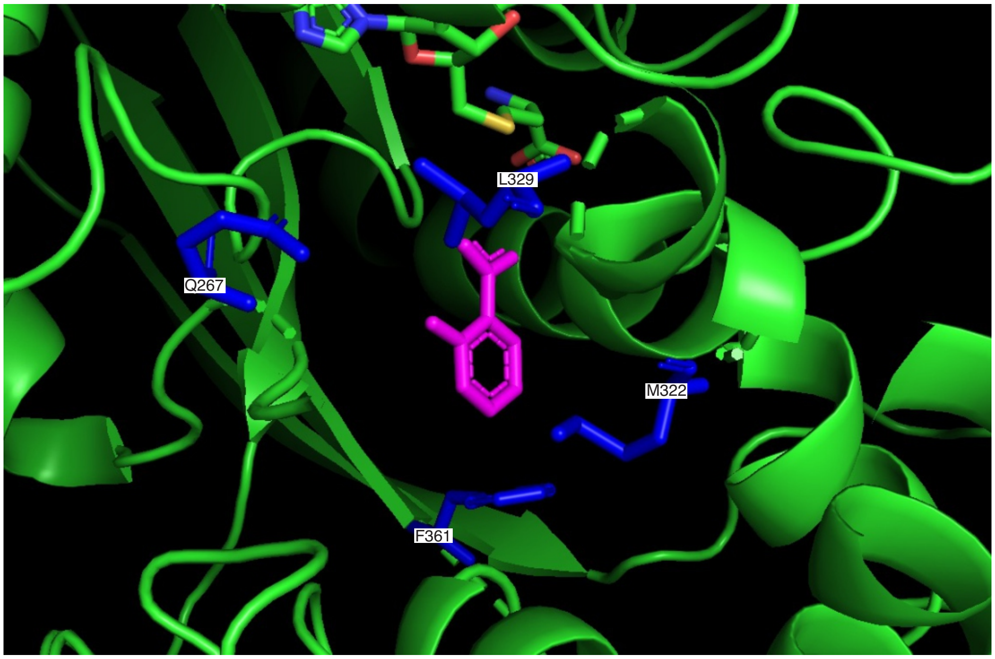

Supplementary Figure S5. Four amino acid positions are positioned close to the substrate bound in the active site of SAMT. These positions also changed during the historical episode when salicylic acid methylation preference appears to have evolved (see Fig. 1 & 2). These sites have remained conserved in the vast majority of angiosperm lineages after the evolution of SA methylation preference (see Fig. 3). All four sites were investigated for their effects on SA preference evolution alone and in all possible combinations as shown in Fig. 4.

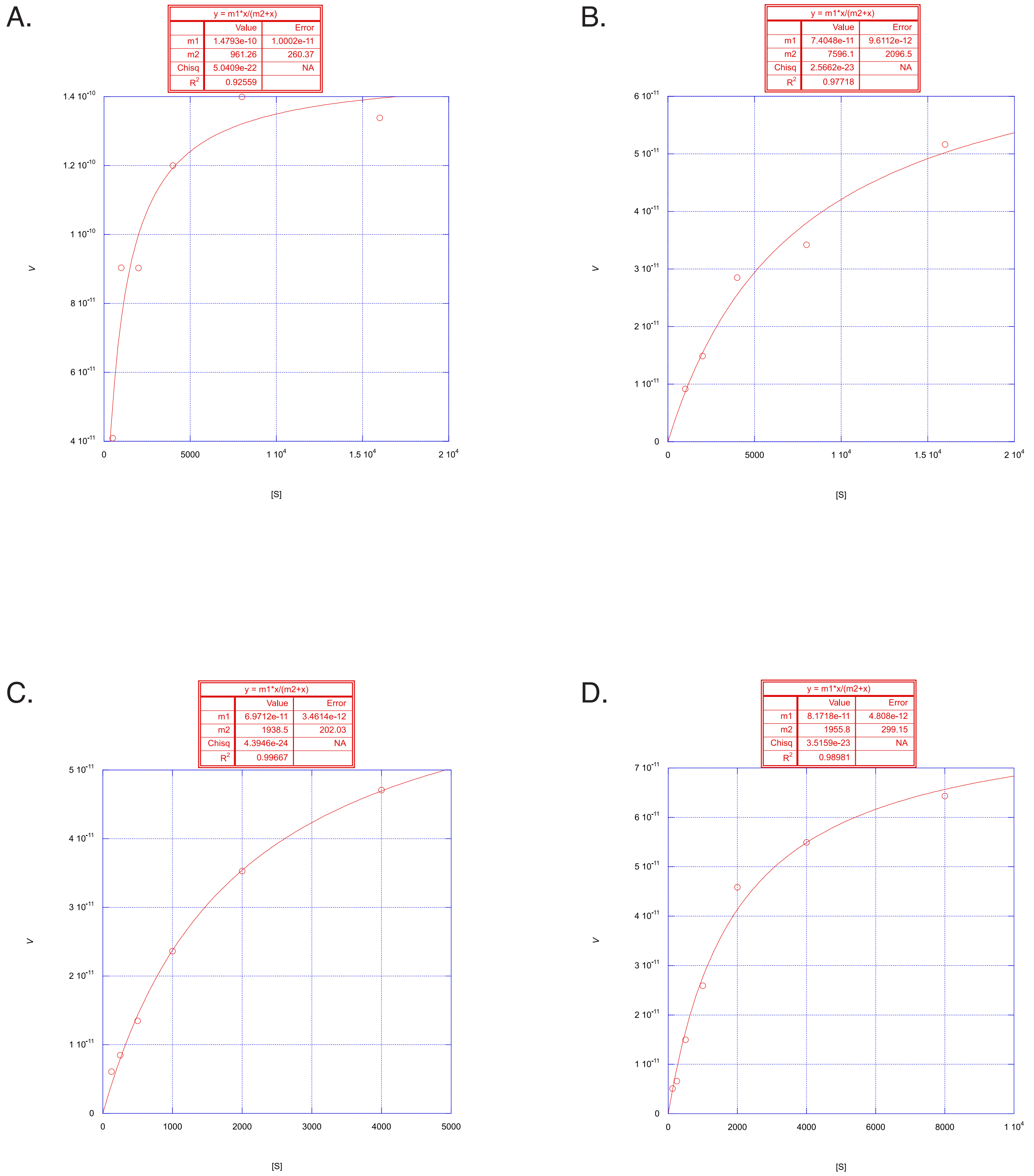

Supplementary Figure S6. Michaelis-Menten kinetics curves fitted to experimental measurements of two ancestral enzymes with two substrates each. A. AncSBXMT + Benzoic acid. B. AncSBXMT + Salicylic acid. C. AncSBXMT Y267Q + Benzoic acid. D. AncSBXMT Y267Q + Salicylic acid. R-squared is shown for each.

Supplementary Table S1. Average and standard deviation of the ratio of methyl salicylate produced relative to methyl benzoate in competitive substrate assays for wild type AncSBXMT and site-directed mutants. A value greater than 1.0 indicates preference to methylate salicylic acid. These data were used in linear regression modeling.

	Ave	SD
AncSBXMT	0.09	0.0004
Y267Q	1.45	0.0079
I322M	0.14	0.0103
M329L	0.18	0.0138
Y361F	0.13	0.0171
Y267Q+M329L	1.97	0.0250
Y267Q+I322M	1.34	0.0951
Y267Q+Y361F	1.12	0.0426
M329L+Y361F	0.11	0.0040
I322M+M329L	0.18	0.0026
I322M+Y361F	0.11	0.0020
Y267Q+I322M+Y361F	1.53	0.1450
Y267Q+M329L+I322M	1.91	0.0702
Y267Q+M329L+Y361F	1.67	0.0426
I322M+M329L+Y361F	0.19	0.0204
Y267Q+I322M+M329L+Y361F	2.83	0.3513

Supplementary Table S2. Primers used for site directed mutagenesis of AncSBXMT.

Mutagenesis Primer Name	Primer Sequence (5'-3')
L238I(F)	gcagttcccatatatagcagcactccttagagga
L238I(R)	tcctctaaggagtgctgctatatatgggaactgc
M329L(F)	gtggctcgccaacaacggttccgcaactg
M329L(R)	cagttgcggaaccgttgttggcgagccac
K348E(F)	catgtgctcagcaacaatctcggcataacggcggaacag
K348E(R)	ctgttccgccgttatgccgagattgttgctgagcacatg
N301D(F)	cgtcgtcgtcgttgtcatttgcgtcccaatttacct
N301D(R)	aggtaaattgggacgcaaatgacaacgacgacgacg
N123D(F)	aacacggaccaaaaccatcgcccttctcttttttcag
N123D(R)	ctgaaaaaagagaagggcgatggttttggtccgtgtt
T54G(F)	caggtcggcgatcccgaggctttccggg
T54G(R)	cccggaaagcctcgggatcgccgacctg
I181M(F)	ggagacgtcttcgccatataaatattacccttgttcaacacg
I181M(R)	cgtgttgaacaagggtaatatttatatggcgaagacgtctcc
A272P_(F)	acctcttccggactcggagcgtaatacggc
A272P_(R)	gccgtattacgctccgagtccggaagaggt
P88S_(F)	acttgaaattccggactcggacggcccagctg
P88S_(R)	cagctgggccgtccgagtccggaatttcaagt
Y267Q_R	cgcactcggagcgtactgcggcaggttgaagct
Y267Q(F)	agcttcaacctgccgcagtacgctccgagtgcg
Y361F(R)	gctgatgaccaggttgatgaatttggtttttctttggacatg
Y361F(F)	catgtccaaagaaaaaccaaattcatcaacctggtcatcagc
I322M(R)	ccgcaactgctctcatgtacttcgccacgttt
I322M(F)	aaacgtggcgaagtacatgagagcagttgcgg
